# Brucellosis in Camels and Humans: Seroprevalence and Associated Risk Factors in Amibara District of Afar Region, Ethiopia

**DOI:** 10.1155/2021/5482725

**Published:** 2021-11-28

**Authors:** Fekadu Gutema Wegi, Kebede Amenu, Adugna Chalchisa, Gezahegne Mamo

**Affiliations:** ^1^Animal Health Research Program, Holeta Agricultural Research Center, Ethiopian Institute of Agricultural Research, P.O. Box 31, Holeta, Ethiopia; ^2^Department of Microbiology, Immunology and Veterinary Public Health, College of Veterinary Medicine and Agriculture, Addis Ababa University, P.O. Box 34, Bishoftu, Ethiopia; ^3^Haramaya University, College of Veterinary Medicine, P.O. Box 138, Haramaya, Ethiopia

## Abstract

Brucellosis is an important neglected zoonotic disease caused by infection with bacteria of the genus Brucella affecting different mammalian species including humans. A cross-sectional study was conducted to estimate the seroprevalence of brucellosis in camels and humans and its associated risk factors in Amibara District of Afar Region in Northeast Ethiopia, from October 2019 to May 2020. A total of 250 camel and 120 human sera were serially tested using the Rose Bengal plate test (RBPT) and complement fixation test (CFT). The overall seroprevalence of camel brucellosis in this study was 7.6% (95% CI: 4.9–11.56) by RBPT and 3.2% (95% CI: 1.63–6.2) by combined RBPT and CFT. In humans, twelve (10%) of the collected sera were positive by RBPT among which only four of them (3.33%) were positive by CFT. The risk factor analysis indicated that age, body condition, number of parity, and abortion history were significantly associated with Brucella seropositivity in camel (*P* ≤ 0.05). In humans, occupation and nonprotective handling of dystocia cases showed an apparent association with Brucella seropositivity. The results of this study indicated that brucellosis is a common health problem in camels and humans in Amibara District of Afar Region. The public health importance of this disease is associated with raw milk consumption and close contact with the animals having history of recent abortion. Therefore, controlling the risk factors, establishing Brucella diagnostic service in human clinics and hospitals, continuous social training with feedback assessments, and overall implementing of One Health approach framework to attain optimal health for people and domestic animals in area are recommended to safeguard the health of society.

## 1. Introduction

Brucellosis is a chronic bacterial disease, which affects numerous domestic and wild mammals with significant public health importance [1]. This disease critically hinders livestock productivity and imposes significant economic loss in the sector [2]. Cattle, camel, sheep, goat, pig, and dog are among the domestic animals that are greatly affected by this disease [3], whereas it is also documented in wildlife and marine animals [4]. The consumption of contaminated fetuses, meat, placentae, or milk is among the major attainment of brucellosis in domestic carnivores [5]. Currently, 11 Brucella species with high genetic similarity and each having different host preferences are recognized [6].

Among these, three Brucella spp., namely, *B. abortus*, *B. melitensis*, and *B. suis,* are known to have high zoonotic and economic importance [6]. Brucella organisms enter into the body of host through inhalation, ingestion, and mucous membrane or broken skin [7]. Brucellosis is found distributed all over the world [8] where greater prevalence is reported from Africa. In Ethiopia, it is found to be one of the endemic diseases of livestock, which is manifested by abortion, late first calving age, long calving interval, low herd fertility, and comparatively low milk production [9, 10]. According to the report of Muma et al. [11] and Schelling et al. [12], cows infected with Brucella are three to four times more expected to abort than the unexposed cows. Besides, this disease posed a barrier to animal transit and largely constrained livestock trade [13].

In humans, brucellosis is a devastating disease that lacks pathognomonic symptoms [14], being a main public health threat, and imposed economic crisis in many countries [15]. Like in domestic and wild mammals, this disease spread among people through the consumption of unpasteurized contaminated milk, raw liver [16], and contact with infected tissues and discharge [17]. Veterinarians, slaughterhouse workers, and laboratory personnel are at great risk of exposure to this chronic disease [18]. Grounded on the nature of the disease, their habit of raw milk consumption, and close physical contact with animals, the pastoralists are at extreme risk of contracting brucellosis [19]. However, since pastoralists usually situate in an inaccessible area, the incidence and the control of brucellosis are ill understood in both humans and animals in the pastoral settings of sub-Saharan Africa [20]. In the pastoral region of Ethiopia, a relatively varying prevalence of brucellosis in both animals and humans has been reported by different authors [21]. According to Ref. [22], the occurrence rates of brucellosis in humans of pastoral and sedentary system origins were estimated at 160 and 28 per 100,000 persons in a year, respectively.

Even though many studies have been made on the seroprevalence of camel and human brucellosis, there is no clear understanding of the geographic pattern of the disease. Additionally, the public health implication of brucellosis in the pastoral areas of Afar Region has not been extensively studied and evaluated by diagnostic techniques rather than relying on survey works [22]. Hence, the availability of recent finding could aid in instituting proper control and prevention measures against this disease for animal owners and communities of the areas at large. Therefore, this study was made to estimate the seroprevalence of brucellosis in camels and exposed individuals and to investigate potential risk factors in Amibara District of Afar Region in Ethiopia.

## 2. Materials and Methods

### 2.1. Description of the Study Area

**Figure 1:**
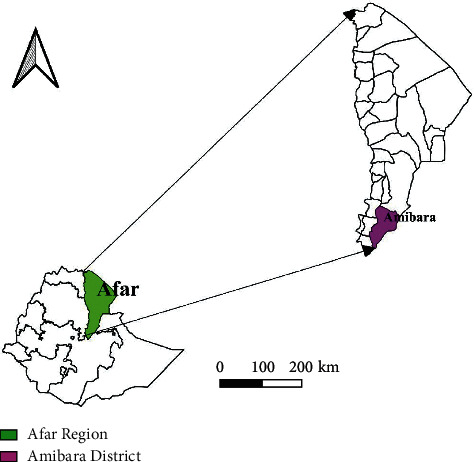
Map showing proposed study district. Source: Diva GIS.

This study was conducted in Amibara District of Gabirasu Zone (Zone 3) of Afar Region in Ethiopia located in the Middle Awash Valley (Figure 1). Amibara District is about 250 km to the Northeast of Addis Ababa and has 19 kebeles with a total population of ∼102,327 of which 56,810 were male and 45,517 are female [23]. The altitude of Amibara District is 740 m above sea level. Monthly based climatic data of fourteen years show that the average maximum and minimum temperature of the area is 34°C and 19°C, respectively, and its annual total rainfall is about 571 mm [24]. The livestock population of the Amibara District is composed of 103, 959 cattle, 122, 526 goats, 48,043 sheep, 3,888 donkeys, and 39,995 camels [25].

### 2.2. Study Population

In the current study, the target study populations were nonvaccinated herds of *Camelus dromedarius* in the hands of pastoralists. Only camels older than six months of age were recruited into the study as the disease was not common in the animals less than 6 months of age. Camels' age was classified into ≤4 yrs, 4–10 yrs, and >10 yrs as young, adult, and old age groups, respectively, according to [26]. To access the magnitude of public health impact, serum samples were also collected from the owners of the sampled camels.

### 2.3. Study Design and Sampling Techniques

A cross-sectional study design was employed from October 2019 to May 2020 in order to determine Brucella seroprevalence in both the camel and the owners of the sampled camel. Study kebeles were chosen purposively based on camel population and accessibility.

To select camel herds in the proposed kebeles, convenience sampling techniques were employed based on the willingness of herd owners to cooperate and accessibility during the period of study. Then, each herd was stratified into subgroups based on age and sex to ensure equal representation of all the subgroups. From each subgroup, individual animals were selected by systematic random sampling technique.

### 2.4. Sample Size Determination of Study Camels

The sample size for serodiagnosis of brucellosis in camel was estimated based on the prior study report in [26, 27] in Afar Region. Hence, the minimum required sample size was calculated using the formula described by [28], with defined 5% precision and 95% confidence level. Thus, by considering prior 4.1% seroprevalence report of camel brucellosis, the minimum required sample size was calculated to be 61 camels. However, to increase precision and minimize standard error, the required sample size obtained by calculation was augmented by fourfold. Hence, about 250 camels were considered for this study from selected kebeles of Amibara District.

### 2.5. Determination of Sample Size of Human Participants

To sample human participants, purposive sampling techniques were employed. Accordingly, all the owners of sampled camels were recruited into the study in which a maximum of four individuals were selected from each household based on their interest. Consequently, 120 individuals were included in this study.

### 2.6. Collection of Blood Sample from Study Camels

Blood samples were collected from each camel preceded by proper restraining to avoid unexpected personal injury and to minimize unnecessary stress that might happen to the animals. After disinfecting the site of jugular vein, 10 ml of blood sample was collected into sterile plain vacutainer tube from each camel. Then, the samples were labeled using code describing herd number, sex, body condition, and kebeles. Then, the samples were taken to laboratory and serum was separated after maintaining at room temperature in slanted position for 24 hrs and centrifuging at 1500 rpm for 5 minutes in WARC. Finally, the serum was gently decanted into sterile cryovials (1.8 ml), labeled, and stored at −20°C until it gets transported to National Veterinary Institute (NVI), Serology Department, Bishoftu, Ethiopia.

In humans, about 5–7 ml of peripheral blood sample was collected from each respondent preceded by verbal agreement. Nurses working in Melka Werer Health Station collected blood samples from the human participants. Then, the sera were separated after allowing to stay in slanted position overnight at room temperature and centrifuging at 1500 rpm for 5 minutes. Finally, the serum from each sample was poured into the cryovials (1.8 ml), labeled, packed, and stored in WARC Animal Health Research Laboratory at −20°C.

### 2.7. Questionnaire Survey

In this study, the owners of the sampled camels and interested individuals were interviewed using a semistructured questionnaire. The questionnaire focused on the knowledge attitude and practice of the society about the zoonotic spread of brucellosis from camels to humans.

### 2.8. Laboratory Diagnosis

#### 2.8.1. Rose Bengal Plate Test

In this study, camel sera were screened for brucellosis by RBPT at the National Veterinary Institute (NVI) of Ethiopia, but human sera were screened by RBPT at WARC and positive to RBPT was further confirmed by CFT at NVI. RBPT was conducted according to the procedures described by the World Organization for Animal Health [29]. *B. abortus* antigen (Lillidale Diagnostics, Holt, Wimborne, Dorset, BH21 7DG, United Kingdom) and their positive and negative control sera were utilized to detect the Brucella antibodies ensuing instructions of the manufacturers. Agglutinations were documented as 0, +, ++, and +++, which designate the absence of agglutination, barely visible agglutination, fine agglutination, and coarse clumping, respectively [30]. The existence of agglutination at any mark was considered as positive reaction, while the absence of agglutination was considered as negative.

#### 2.8.2. Complement Fixation Test (CFT)

Serum samples reactive to RBPT were further tested by CFT for confirmation using standard *B. abortus* antigen S99 (New Haw, Addlestone, Surrey, KTI5, and 3NB-UK). The preparation of the reagent is evaluated by titration and was performed according to the protocol recommended by World Organization for Animal Health [31]. Sera with more than 75% fixation of complement (3+) at a dilution of 1 : 5 or at least with 50% fixation of complement (2+) at a dilution of 1 : 10 and above were considered as positive result, whereas the lack of fixation/complete hemolysis was considered as negative result. Only samples that gave signals for both RBPT and CFT were considered positive since no single test is appropriate in all epidemiological situations due to problems of sensitivity and or specificity of the tests as recommended by OIE and other reports [32].

### 2.9. Data Analysis

Camel and human parameters, serological results, and questionnaire data were recorded in a Microsoft Excel® Spreadsheet, and the analysis was carried out using R Software version 4.0.0. Prevalence was calculated by dividing the number of positive animals and humans to the total number of animals and humans tested. Fisher's exact test was used to calculate the associations of risk factors with Brucella seropositivity. In this study, the numbers of outcomes of interest were less than 10% of the total sample size of camels and humans. Thus, Firth's bias-reduced logistic regression model was used to measure the association of potential risk factors with Brucella seropositivity [33]. Odds ratio (OR) was used to measure the degree of association between the risk factors such as herd size, age, sex, body condition, parity number, abortion history, and RFM with animal-level seroprevalence. *P* value less than 0.05 was considered statistically significant in all analyses.

### 2.10. Ethical Consideration

The research ethical clearance for gathering of animal materials was approved by the Animal Research Ethical Review Committee of the College of Veterinary Medicine and Agriculture (CVMA) with certificate reference number of VM/ERC/03/01/12/2020. Pastoralists were also informed of the aim of the study, and their agreement was sought before the commencement of questionnaire data collection. For the collection of blood samples from the human participants, the work had earned recognition and written consent was obtained from Afar Regional Health Bureau by reference number of QAPB011/3934.

## 3. Result

### 3.1. Seroprevalence of Camel Brucellosis and Associated Risk Factors

Of the 250 camel samples tested, 19 were positive by RBPT and 12 were confirmed by CFT. Thus, the overall seroprevalence of camel brucellosis in this study is 7.6% (95% CI: 0.049–0.1156) by RBPT and 3.2% (95% CI: 0.0163–0.062) by combined RBPT and CFT. In Table 1, sex-wise brucellosis seroprevalence and association of abortion with Brucella infection in camel were indicated. Accordingly, abortion history showed a statistically significant association with Brucella seropositivity (*P* ≤ 0.01^*∗*^), which means abortion in camels is mainly due to brucellosis. It was also indicated that female animals with abortion history were 36.2 times more tested positive for brucellosis than nonaborted animals (OR = 36.2, 95% CI = 7.52–351.9).

The association of possible risk factors with Brucella seropositivity in camel was indicated (Table 2). Consequently, no statistically significant difference in Brucella seropositivity was observed between both sexes of camel (*P* > 0.05). Additionally, age (*P* ≤ 0.004), history of abortion (*P* ≤ 0.005), body condition (*P* ≤ 0.003), and number of parity (*P* ≤ 0.001) were found to be significantly associated with Brucella seropositivity in camel. However, herd size and placental retention were considered and did not show significant association with brucellosis in camels (*P* > 0.05).

### 3.2. Multivariable Analysis of Risk Factors Associated with Brucellosis in Camel

Grounded on the multivariable Firth's bias-reduced logistic regression analysis, only history of abortion is independently associated with brucellosis in camel (*P* ≤ 0.002). Briefly, camels having history of abortion were 49.6 times more tested positive for brucellosis than those with no history of abortion. Camels with number of parity greater than 3 were also 2.75 times more at risk of contracting brucellosis than young camels (Table 3).

### 3.3. Serological Results of Human Brucellosis

Of 120 human sera tested by RBPT, 12 were found reactive among which only 4 confirmed by CFT to be Brucella seropositive. In this study, the occupation of the study participant showed a statistically significant association with Brucella seropositivity in humans (*P* ≤ 0.03) based on Fisher's exact test (Table 4).

### 3.4. Multivariable Analysis of Potential Risk Factors Associated with Brucella Seroprevalence in Humans

Following computation of univariable Firth's bias-reduced logistic regression analysis, all sociodemographic risk factors were insignificantly associated with Brucella seropositivity in humans. Moreover, individuals with nonpermanent work were 18.8 times more at risk of contracting brucellosis when compared with government employee and also showed a statistically significant association (*P* ≤ 0.03^*∗*^, 95% CI: 1.324, 2730.32) with Brucella infection (Table 5).

## 4. Results of Questionnaire Survey

Questionnaires were administered to all 120 participants to gather information vis-à-vis their knowledge about zoonotic diseases and management of their livestock. Consequently, 80% of the respondents have no information of disease transmission from wild to domestic animals (Table 6). Moreover, 76.47% of the respondents were found to manage their animals by mixing different species, whereas the majority of them keep their animals in national park and practice inappropriate disposal of the aborted fetus and placental membrane, which are the major predisposing factors for the occurrence of brucellosis in cattle and camel (Table 6).

Moreover, consumption of raw meat and milk, knowledge of brucellosis, and other zoonotic diseases were considered and did not stand significantly (*P* > 0.05) with Brucella seropositivity in humans. Even though the statistical significance was not met, all seropositive individuals were consumers of raw milk, which elucidated that raw milk consumption is more associated with Brucella infection in the area. In this study, 90% of the respondents did not know about brucellosis and it comprises 10 positive animals and all reactive individuals. In this study, 91.7% of the respondents drink raw milk among which 3.63% of them tested positive for brucellosis (Table 7).

## 5. Discussion

Brucellosis is an infectious bacterial disease affecting all domestic and wild animals with significant economic and public health importance. In this study, the animal-based seroprevalence of camel brucellosis in Amibara District of Afar Region was 7.6% by RBPT and 3.2% by combined RBPT and CFT. Thus, this study revealed that the overall seroprevalence of camel brucellosis was 3.2% (8/250).

This finding is comparable with the former reports of 3.1% in Yabello District of Borena Zone by Admasu et al. [34], 3.37% in Mehoni District of Southeastern Tigray by Habtamu et al. [35], and 3% in Southern lowland of Ethiopia by Jara et al. [36], but it is lower when compared to 5.7% in three camel rearing regions (Afar, Somali, and Borena) by Teshome et al. [37], 7.6% in Awash Fentale and Amibara districts of Afar Region by Zewolda and Wereta [38], and 5.4% in four districts of Afar regional state by Bekele et al. [39]. This study result is also lower than some reports in other African and Middle East countries when compared with a prevalence of 30.5% in Sudan by Mokhtar et al. [40], 7.61% in Egypt by Hassanain and Ahmed [41], and 19.4% in Jordan by Dawood [42]. 21.74% (5/23) herd-level seroprevalence of camel brucellosis was recorded in this study, which is similar to the reports of [27] in Afar Region of Northeastern Ethiopia.

In this study, a higher number of Brucella seropositivity was seen in adults and older camels than in younger animals as it is a disease of sexually matured and pregnant animals, which agrees with the findings of Ref. [9]. The increased incidence of brucellosis in pregnant and sexually matured animals is associated with increased production of erythritol sugar at this stage of life, which enhances the multiplication of pathogen [26].

Herd size of the camels was also considered in this study to see the dynamics of this disease in different herd groups since it is known to be the disease of herd importance. However, no statistically significant difference (*P* > 0.05) in Brucella seropositivity was observed among different herd groups of camels based on Fisher's exact test even though this finding disagrees with the report of Ref. [26].

To boost the immunity of the host against various infectious diseases, diet plays a decisive role. Malnourished animals are likely to have poor body condition, which is revealed by decreased immunity, persistent occurrence of infection, and vulnerability to noninfectious organisms under normal condition [43]; [44]. Taking this into account, body condition of the camels was considered to see the status of Brucella infection in different body condition scores. As a result, statistically significant higher seropositivity of brucellosis was observed in camels with poor body condition score than in camels with medium or good body condition score (*P* ≤ 0.01), whereas similar findings were also reported by Abebe et al. [9].

Human brucellosis is a more prevalent disease in the pastoral areas of different African and Asian countries [19]. In this study, an overall human Brucella seropositivity of 3.33% was identified by combined RBPT and CFT. This finding is fairly in agreement with the findings of Ref. [45] in Jimma University Hospital, [46] in Addis Ababa, [47] in Eastern Ethiopia, and [48] in Adami Tullu who reported 3.6%, 4.8%, 4.8%, and 2.15%, respectively. However, the result of this study is lower than the finding of Ref. [49] in Turkey and Ref. [50] in India who reported 5.4% and 4.96%, respectively.

However, it is higher than the finding of Ref. [51] in Bangladesh and Ref. [52] in western who reported 2.0% and 1.2%, respectively. In the pastoral and agropastoral areas, the prevalence of brucellosis in humans is greatly influenced by the status of the disease in animals [53]. Brucellosis occurrence significantly varies extensively between and with countries [54]. So, the possible justification for the variation of the current human Brucella seroprevalence from the previous report was due to the difference in the sample size, difference in the lifestyle of the society, type of diagnostic protocol employed, and socioeconomic status of the study population.

One research work conducted in Malaysia showed that males are at greater risk of contracting brucellosis since they are commonly involved in the handling of livestock and consume uncooked animal product, especially in the pastoral area [55]. However, the finding of this study indicated that gender is not significantly associated with brucellosis seropositivity and did not much with the report of the above research finding in Malaysia. This may be due to small sample size of this study and shared responsibility among males and females of Afar pastoralists. More than 90% of people in Afar Region depend on pastoral activity, and they spend almost all their entire life with their animals. The main source of their food is also animals' origin, especially milk, which is commonly consumed without boiling. In this study, 96.4% of the respondents drink raw milk, which agrees with the findings of Ref. [56] who reported that the consumption of uncooked food plays a crucial role in the transmission of brucellosis in humans. Nonsignificant association of educational background with brucellosis seropositivity of the respondent was found in this study (*P* > 0.05) because of low basic infrastructure in the area aligned with mobile lifestyle of the society.

However, the study conducted in Kenya showed a high level of knowledge of brucellosis in pastoral communities where respondents testified brucellosis to be a zoonotic disease and abortion as its common indicator [57]. This might be due to the difference in the educational access and coverage in the pastoral area of the two countries. Even though the association between raw milk consumption and Brucella seropositivity is statistically not significant, all of the seropositive individuals were consumers of raw milk. 78.33% (*n* = 94) of this study's participants told that they do not consume raw meat, which is mainly concerned with cultural issue. This shows that the rate of Brucella infection in this study area is greatly associated with drinking of raw milk and contact with infected animals compared with the consumption of uncooked meat.

During handling of dystocia cases, the majority of the respondents (98.2%) reported that they used hand pulling techniques without using protective gloves (90%). The study conducted by Eshetu et al. [47] indicated that brucellosis was 5.11 times more in those who had assisted animals during parturition than in those who did not. Regarding the management of aborted fetal membrane/aborted fetus and discharge, 83 (81.37%) of the respondents told that they throw it on the field. Additionally, it was also encountered that pastoralists leave dead camel calf at home to show the mother camel every morning by considering that the activity will instinct milk letdown. So, these activities can be the major predisposing factors for the widespread occurrence of brucellosis in camels, other domestic animals, and owners. However, 4.9% of the respondents practiced proper disposal of the aborted fetal materials. In this study, about 90% of the respondents had never heard about brucellosis and 65% of them need to acquire detailed information about it. Finally, the major symptoms of brucellosis were reported to respondents and about 99.1% of them were interested in giving blood samples for screening of brucellosis.

## 6. Conclusion and Recommendations

Brucellosis is one of the most vital bacterial diseases of domestic and wild animals with substantial economic and public health importance. Both animals and humans can contract brucellosis through direct contact with infected animals and their excreta, ingestion of infected materials, and sometimes through aerosol transmission. The results of this study indicated that brucellosis is a common health problem in camels and humans in Amibara District of Afar Region. The public health importance of this disease is associated with raw milk consumption and close contact with the animals having history of recent abortion.

Therefore, based on the present finding, the following recommendations are worth mentioning:

- Requiring a comprehensive active assessment and surveillance studies to understand the distribution of brucellosis and its transmission dynamics at domestic-wild life interface and its zoonotic significance
- Working to control risk factors, establishing Brucella diagnostic service in human clinics and hospitals, and implementing One Health approach framework to attain optimal health for people and animals
- Enhancing the awareness level of the pastoral society about the public health and economic significance of brucellosis through training and workshop
- Conducting detailed studies on isolation and characterization of circulating strain and biotypes in the area

## Figures and Tables

**Table 1:** Overall Brucella seroprevalence and association of abortion with the disease in camel.

Variables	Number tested	No. of RBPT positive (%)	No. of CFT positive (%)	*P* value	OR (95% CI)
*Sex*				0.814	0.7 (0.075–91.85)
Male	9	1	0 (0)		
Female	223	18	8 (3.2)		

*Abortion history* ^*∗∗*^				≤0.01^*∗*^	36.2 (7.52–351.9)
Yes	37	15 (42.1)	7 (19.23)		
No	204	4 (3.4)	1 (0.284)		

^*∗∗*^Only female animals were considered; ^*∗*^significant.

**Table 2:** Summary of Brucella seropositivity in camels and association of potential risk factors.

Variables	Number tested	Seropositive	Prevalence (%)	*χ* ^2^	*P* value
*Sex*				0.309^a^	1
Male	9	0	0		
Female	241	8	3.43		

*Age*				10.7^b^	≤0.004^*∗*^
Young	34	0	0		
Adult	165	2	1.21		
Old	51	6	11.764		

*Body condition*				10.26^b^	≤0.003^*∗*^
Poor	53	6	11.3		
Medium	158	2	1.266		
Good	39	0	0		

*Herd size*				0.926^b^	0.6357
<20	42	2	4.76		
20–50	92	2	2.17		
>50	116	4	3.45		

*Number of parity* ^*∗∗*^				11.2^b^	≤0.001^*∗*^
Null	32	0	0		
Less than or = 3	160	2	1.25		
Greater than 3	49	6	12.24		

*Abortion history* ^*∗∗*^				29.96^a^	≤0.005^*∗*^
Aborted	37	7	18.92		
No abortion	213	1	0.47		

*Placental retention* ^*∗∗*^				0.134^b^	1
Yes	4	0	0		
No	229	8	3.5		

^b^Fisher's exact test value; ^a^ chi-square value; ^*∗*^significant; ^*∗∗*^only female animals considered.

**Table 3:** Multivariable Firth's bias-reduced logistic regression analysis of factors associated with brucellosis seropositivity in camel.

Variables	Number tested	Seropositive	Adjusted OR	(95% CI)	*P* value
*Parity number*					
Null	32	0	1	1	—
Less than or = 3	160	2	0.15	(−5.31, 3.233)	0.357
Greater than 3	49	6	2.75	(−195, 6.00)	0.511

*Abortion history*					
Yes	36	7	49.6	(2.148, 6.34)	≤0.002^*∗*^
No	205	1		1	—

1: reference

**Table 4:** Sociodemographic characteristics and Brucella seroprevalence among the study participants.

Variables	No. of tested *N* (%)	RBPT + ve		CFT + ve		*χ* ^2^	*P* value
		*n*	%	*n*	%		
*Gender*							0.19
Male	95 (79.2)	8	8.4	2	2.1		
Female	25 (20.8)	4	16	2	8		

*Age*						5^b^	0.057^.^
13–19 years	6 (5)	1	16.67	1	16.67		
20–59 years	79 (65.8)	6	7.6	1	1.26		
Above 60 years	35 (29.17)	5	14.3	2	5.71		

*Educational status*						3.7^b^	0.786
Degree	1 (0.83)	0	0.0	0	0.0		
Diploma	3 (2.5)	0	0.0	0	0.0		
High school	10 (8.33)	0	0.0	0	0.0		
Elementary	16 (13.34)	2	12.5	1	6.25		
Read and write	9 (7.5)	1	11.1	0	0.0		
Illiterate	81 (67.5)	9	11.1	3	3.7		

*Occupation*						6.925^b^	≤0.03^*∗*^
Pastoralists	90 (75)	7	7.78	2	2.22		
Government	22 (18.34)	2	9.09	0	0.0		
Others	8 (6.67)	3	37.5	2	25		

*Marriage*						2.6^b^	0.484
Married	102 (85%)	11	10.78	3	2.94		
Divorced	1 (0.833)	0	0.0	0	0.0		
Single	17 (14.17)	1	5.88	1	5.88		

*Family size*						1.694^b^	0.375
1–2	30 (25)	4	13.33	2	6.67		
3–5	58 (48.33)	6	10.34	1	1.72		
Above 6	32 (26.67)	2	6.25	1	3.12		

^b^Fisher's exact test value; No. of tested = number of tested individuals.

**Table 5:** Multivariable Firth's bias-reduced logistic regression analysis of factors associated with brucellosis seropositivity in humans.

Variables	No. of tested	Seropositive	Adjusted OR	(95% CI)	*P* value
*Occupation*					
Government	22	0	1	1	—
Others	8	2	18.85	(1.324, 2730.32)	≤0.03^*∗*^
Pastoralists	90	2	1.42	(0.11, 197.86)	0.817

No. of tested = number of individuals tested; OR=odds ratio; 1: reference.

**Table 6:** Zoonotic disease knowledge and livestock keeping practice of pastoralists in the study area.

Variables	Response category	Frequency	%
*Do you think that diseases can get transmitted from animals to humans?*			
	Yes	24	20
	No	96	80

*How do you herd your animals?*	(*N* = 102)		
	Mix at gazing point	78	76.47
	Mix at watering point	7	6.86
	All separately	22	21.57
	All together	13	12.74

*Do you keep animals in national park?* ^*∗*^	(*N* = 90)		
	Yes	80	88.89
	No	10	11.11

*Frequent occurrence of abortion* ^*∗∗*^	(*N* = 102)		
	Yes	68	66.67
	No	34	33.33

*Which animals frequently abort?* ^*∗∗*^	(*N* = 102)		
	Cattle	15	14.7
	Camel	9	8.82
	Small ruminant	54	52.94
	No animal abort	24	23.53

*Disposal of placental membrane and aborted fetus*	(*N* = 102)		
	Dispose properly	5	4.9
	Throw it on open field	83	81.37
	Report to CAHWS	14	13.72

^*∗*^Only pastoralists considered; ^*∗∗*^only respondents having animals considered.

**Table 7:** Association of knowledge and practice of the camel owners with Brucella seropositivity.

Variables	Respondents	Positive animals owned	Positive respondents	Fisher's value	*P* value
	n (%)	(%)	n (%)		
*Do you drink raw milk?*				—	1
Yes	110 (91.7)	12 (2.79)	4 (3.63)		
No	10 (8.3)	0 (0)	0 (0)		

*Do you consume raw meat?*				—	0.572
Yes	27 (22.5)	3 (0.69)	1 (3.7)		
No	94 (78.33)	9 (2.08)	3 (3.2)		

*Do you know zoonotic diseases?*				—	0.318
Yes	34 (28.33)	8 (1.85)	2 (5.88)		
No	86 (71.67)	4 (0.93)	2 (2.32)		

*Do you know brucellosis?*				—	1
Yes	12 (10)	2 (0.46)	0 (0.0)		
No	108 (90)	10 (2.32)	4 (3.7)		

*Do you use PG during handling dystocia case?*				—	≤0.05^*∗*^
Yes	12 (10)	2 (0.46)	2 (16.67)		
No	108 (90)	10 (2.32)	2 (1.85)		

*How do you shelter camels?*				9.08^b^	≤0.03^*∗*^
All separately	82 (68.33)	10 (2.32)	1 (1.22)		
All sheltered together	2 (1.67)	0 (0.0)	1 (50)		
Some sheltered together	24 (20)	2 (0.46)	2 (8.33)		
No idea	12 (10)	0 (0)	0 (0)		

CAHWs: community-based animal health workers; ^b^Fisher's exact test value; ^a^ chi-square value; ^*∗*^significant.

## Data Availability

The data used to support the findings of this study are available from the corresponding author upon request.
